# Prolidase deficiency, a rare inborn error of immunity, clinical phenotypes, immunological features, and proposed treatments in twins

**DOI:** 10.1186/s13223-022-00658-2

**Published:** 2022-02-23

**Authors:** Nora Alrumayyan, Drew Slauenwhite, Sarah M. McAlpine, Sarah Roberts, Thomas B. Issekutz, Adam M. Huber, Zaiping Liu, Beata Derfalvi

**Affiliations:** 1 Division of Immunology, Department of Paediatrics, Dalhousie University, IWK Health Centre, Halifax, Canada; 2 Division of Rheumatology, Department of Paediatrics, Dalhousie University, IWK Health Centre, Halifax, Canada; 3 Division of Clinical Biochemistry & Maritime Newborn Screening, Department of Pathology and Laboratory Medicine, Dalhousie University, IWK Health Centre, Halifax, Canada

**Keywords:** Prolidase deficiency, T cells, Inborn error of immunity, Autoimmunity, Leg ulcers

## Abstract

**Background:**

Prolidase deficiency (PD) is an autosomal recessive inborn multisystemic disease caused by mutations in the *PEPD* gene encoding the enzyme prolidase D, leading to defects in turnover of proline-containing proteins, such as collagen. PD is categorized as a metabolic disease, but also as an inborn error of immunity. PD presents with a range of findings including dysmorphic features, intellectual disabilities, recurrent infections, intractable skin ulceration, autoimmunity, and splenomegaly. Despite symptoms of immune dysregulation, only very limited immunologic assessments have been reported and standard therapies for PD have not been described. We report twin females with PD, including comprehensive immunologic profiles and treatment modalities used.

**Case presentation:**

Patient 1 had recurrent infections in childhood. At age 13, she presented with telangiectasia, followed by painful, refractory skin ulcerations on her lower limbs, where skin biopsy excluded vasculitis. She had typical dysmorphic features of PD. Next-generation sequencing revealed pathogenic compound heterozygous mutations (premature stop codons) in the *PEPD* gene. Patient 2 had the same mutations, typical PD facial features, atopy, and telangiectasias, but no skin ulceration. Both patients had imidodipeptiduria. Lymphocyte subset analysis revealed low-normal frequency of T_reg_ cells and decreased frequency of expression of the checkpoint molecule CTLA-4 in CD4^+^ T_EM_ cells. Analysis of Th1, Th2, and Th17 profiles revealed increased inflammatory IL-17^+^ CD8^+^ T_EM_ cells in both patients and overexpression of the activation marker HLA-DR on CD4^+^ T_EM_ cells, reflecting a highly activated proinflammatory state. Neither PD patient had specific antibody deficiencies despite low CD4^+^CXCR5^+^ T_fh_ cells and low class-switched memory B cells. Plasma IL-18 levels were exceptionally high.

**Conclusions:**

Immunologic abnormalities including skewed frequencies of activated inflammatory CD4^+^ and CD8^+^ T_EM_ cells, decreased CTLA-4 expression, and defects in memory B cells may be a feature of immune dysregulation associated with PD; however, a larger sample size is required to validate these findings. The high IL-18 plasma levels suggest underlying autoinflammatory processes.

## Background

Prolidase deficiency (PD) (OMIM170100) is an autosomal recessive inborn error of metabolism caused by mutations in the *PEPD* gene encoding the enzyme prolidase D, leading to defects in turnover of collagen and other proline-containing proteins. Since it was first described in 1968 by Goodman [1], 35 causative mutant alleles have been reported in the ninety cases worldwide [2, 3]. The incidence of PD is estimated to be 1–2 per million births, but it is more frequent in some populations, including the Druze and Arab Muslim minorities in Israel [2–6]. PD is categorized under Diseases of Immune Dysregulation, Syndromes with Autoimmunity in the 2019 IUIS Phenotypical Classification of Human Inborn Errors of Immunity [7]. PD is often referred to as a “lupus mimic”, presenting predominantly with cutaneous features and with systemic autoimmunity [8–12]. Typical presentation of PD includes intractable skin ulceration, telangiectasia, recurrent infections, splenomegaly, dysmorphic features, and intellectual disabilities [2, 13]. Thrombocytopenia, hypocomplementemia, and hypergammaglobulinemia are frequent laboratory findings, as are increased levels of proline metabolites in serum and urine [9, 14–18].

To date, no standard diagnostic criteria or testing algorithms for PD have been published. PD is diagnosed by the detection of either biallelic *PEPD* pathogenic variants or reduced prolidase enzyme activity in patients who present with characteristic clinical findings and imidodipeptiduria [2, 17, 19]. The aim of this study was to identify features associated with immune dysregulation in PD patients through comprehensive immunologic assessments.

## Case presentation

The index case, Patient 1 (Pt1), had recurrent ear and throat infections in early childhood, which when treated with antibiotics, tonsillectomy, adenoidectomy, and bilateral myringotomy tube insertion, lead to less frequent infections. At 13 years of age, Pt1 presented with severe, chronic, refractory skin ulcerations on her lower extremities, which prevented ambulation. Patient 2 (Pt2), an identical twin of Pt1, presented with recurrent respiratory tract infections as well as severe atopy, including anaphylaxis to multiple food allergens, allergic rhinitis, and asthma. Pt2 also had recurrent arthralgia, mainly in the large joints, with no stiffness or limitation of movement. No skin ulceration was present in Pt2. Both Pt1 and Pt2 presented with telangiectasia predominantly in their lower extremities. The clinical findings of both patients are summarized in Table 1.

**Table 1:** Clinical presentation in twin females with PD

Known clinical presentation of PD	Patient 1	Patient 2
Skin manifestations	Diffuse telangiectasias Ulceration of the feet	Telangiectasias one foot ulcer after 2 years
Dysmorphic features	+	+
Intellectual disability (ID)	+	+
Recurrent infections	+	+
Coexistence autoimmunity	Euthyroid autoimmune thyroiditis	−
Allergic symptoms and atopy	−	++ Bronchial asthma, allergic rhinitis, eczema, food allergy
Endocrinopathies	−	−
Pulmonary manifestations	−	−
Splenomegaly/hepatomegaly	+ / +	−/ +
Other		Arthralgia and hyperlaxity

+ , clinical feature present; −, clinical feature absent

**Fig. 1:**
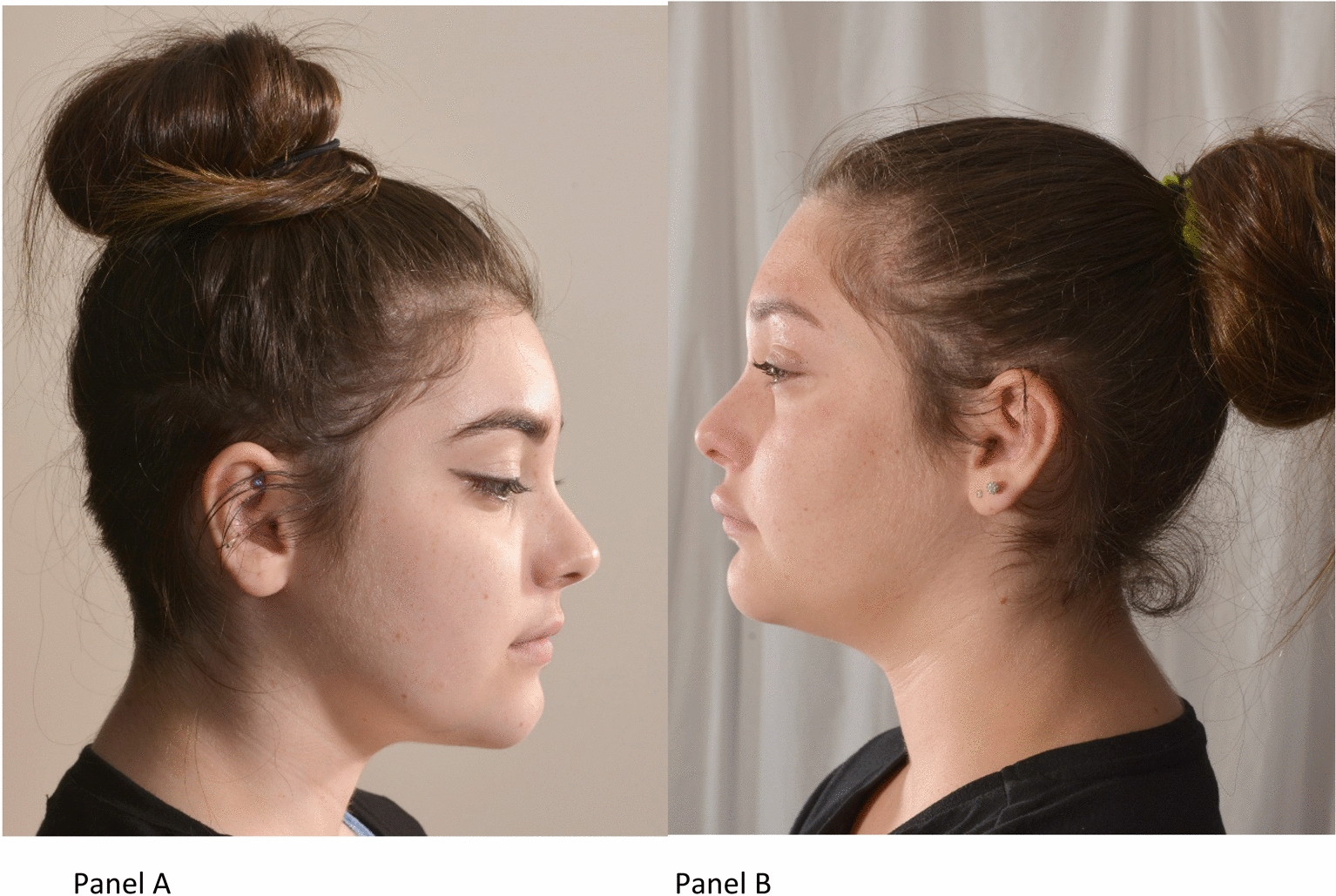
Dysmorphic facial features in Patient 1 (Panel A) and patient 2 (Panel B) include low hairline, mild ptosis, hypertelorism, depressed nasal root, beak-like nose, and micrognathia

**Fig. 2:**
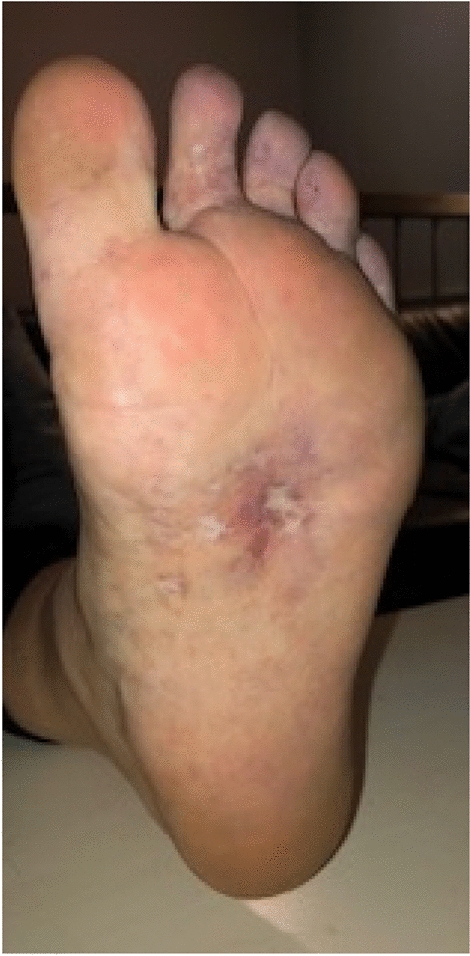
Multiple ulcers at different stages of healing on the foot of Patient 1

**Fig. 3:**
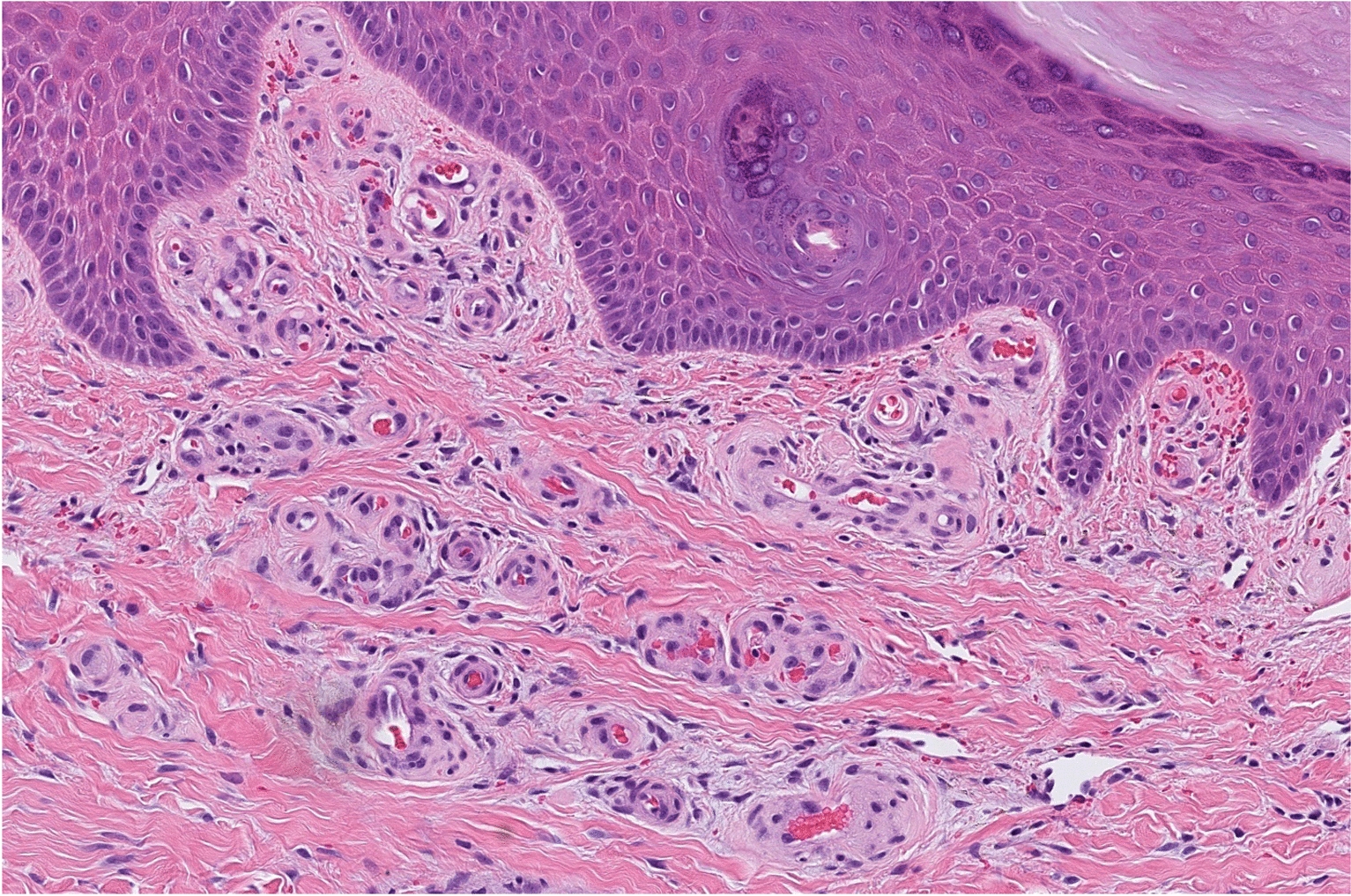
Skin punch biopsy (sole) in Patient 1. Lobulated proliferation of capillaries, hemorrhage in dermis. The overlying epidermis is normal. No vasculitis. *Hematoxylin–Eosin* staining, × 400

**Fig. 4:**
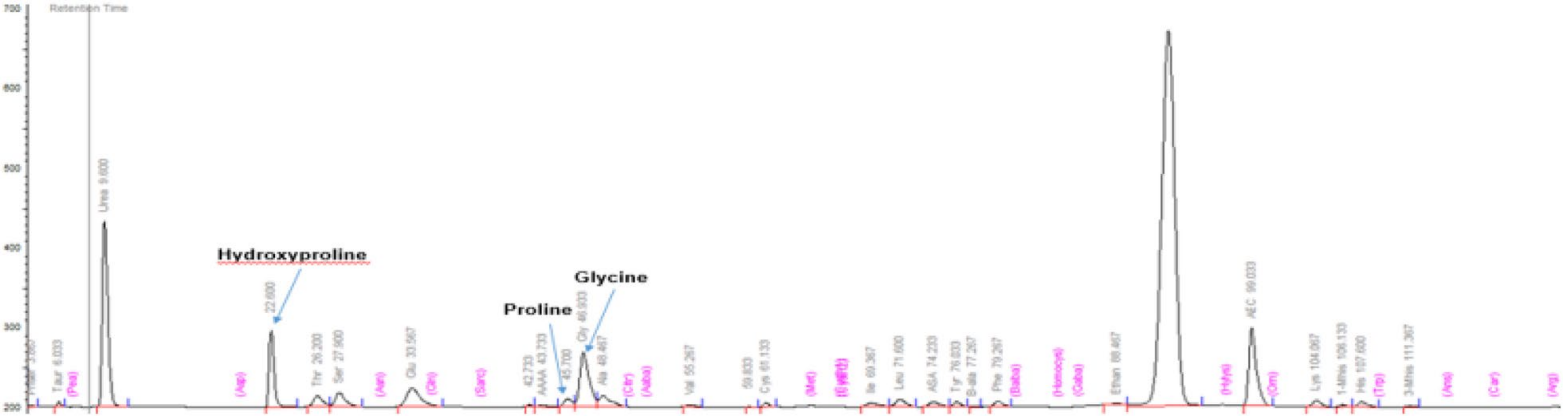
Imidodipeptiduria, shown by increased Proline, Hydroxyproline and Glycine on urine amino acid quantitation by Ion-Exchange Chromatography (Biochrom amino acid analyzer)

Physical examination at 15 years of age showed that both twins had dysmorphic features, including a low hairline, mild ptosis, hypertelorism, a depressed nasal root, a beak-like nose, and micrognathia (Fig. 1). Pt1 had ulcers on the feet at different stages of healing with white discoloration (“atrophie blanche”) of the skin around the ulceration (Fig. 2). Pt1 also had splenomegaly. Musculoskeletal examination was normal in both cases, except for observed hypermobility in Pt2. A sole skin punch biopsy was performed for Pt1 and excluded vasculitis but revealed livedoid vasculopathy with perivascular lymphocytic infiltration (Fig. 3). Poor wound healing was observed as the biopsy site did not heal for one year. Pt1 was diagnosed with PD at the age of 15 following next generation sequencing of 298 primary immunodeficiency genes (Blueprint Genetics). Previously reported pathogenic compound heterozygous mutations (c.977G>A, p.Trp326* and c.550C>T; p.Arg184*), which introduced premature stop codons in the *PEPD* gene, were detected [20, 21]. Pt2 was found to have the same mutations. The non-consanguineous asymptomatic parents are carriers of one of the *PEPD* mutant variants, confirming trans position of the mutated alleles. Imidodipeptiduria, indicating increased proline metabolites, confirmed PD in both patients [22] (Fig. 4). Family history for immune-mediated diseases was unremarkable except for maternal psoriasis and recurrent streptococcal infections.

Pt1 required gabapentin for pain control and antidepressants to treat severe anxiety, self-harm, and depression. She was started on low-molecular-weight heparin (LMWH), pentoxifylline to improve microcirculation, and high dose (1 g/kg) intravenous immunoglobulin (IVIG) infusions for immune modulation. This resulted in the cessation of disease progression and partial healing of ulcers. After genetic diagnosis, more targeted treatments were added, including high-dose collagen synthesis cofactor vitamin C (500 mg/day), 5% proline–glycine ointment, and topical tacrolimus (0.33%). Pt1 responded well to this combined therapy initially, allowing for recovery of full physical activity, and showed no new ulceration for 4 months. All previous ulcerations closed, leaving scars and whitish discoloration. Pt1 subsequently developed one new foot ulcer, which did not impact mobility and began to heal more quickly than previous ulcers.

Pt2 was started on high dose vitamin C and pentoxifylline after presenting with white skin atrophy on the feet and mutation within *PEPD* was confirmed. One year later, Pt2 developed a small ulceration which was treated immediately with topical tacrolimus (0.33%) and 5% proline–glycine ointment, which stopped ulcer progression and promoted healing.

Magnetic resonance imaging (MRI) of the brain was performed for Pt1 due to chronic headaches, to rule out vasculitis, with normal results. Abdominal ultrasound showed splenomegaly in Pt1, which has been previously reported in PD [2, 3, 23], as well as hepatomegaly and diffuse hepatic steatosis in Pt1 and Pt2. The serum lipid profile revealed elevated low-density lipoprotein (LDL) and triglyceride levels in both patients, which has not been reported previously in PD. Pt1 had an overall low Wechsler Intelligence Scale for children—5th edition (IPad) (WISC-V) IQ score at the 5th percentile when tested at age 16, and she performed at the level of a 12-year-old on the vocabulary test. The WISC-V IQ score of Pt2 ranged from extremely low to low average in the various test components.

A detailed laboratory assessment was conducted including measurement of humoral immunity, three pathways of complement activation, T and B lymphocyte proliferation to mitogens and antigens, and natural killer (NK) cell cytotoxicity. Findings of laboratory investigations are detailed in Tables 2 and 3. A complete blood count showed mild thrombocytopenia in Pt1. Immunoglobulin (total IgG, IgG1-4, IgA, IgM, IgE) levels were normal with the exception of low IgG2 in Pt2. Total B cell counts were normal and antibody titers to tested vaccine antigens were sustained as protective, confirming normal specific antibody production, despite both patients having low peripheral class-switched memory B cells. Lymphocyte proliferation in response to stimulation with mitogens and antigens was normal as detected via H^3^-thymidine incorporation assay. NK cell cytotoxicity, indicated by degranulation and target cell killing, was also normal. Both patients were mannose-binding lectin (MBL) deficient. Autoantibody measurements confirmed antinuclear antibody (ANA) and anti-thyroid peroxidase (TPO) antibody positivity in Pt1. Blood inflammatory parameters C-reactive protein (CRP) and erythrocyte sedimentation rate (ESR) were elevated in Pt1, even when the patient’s ulcers resolved.

**Table 2:** Clinical laboratory results in twin females with PD

Test	Patient 1	Patient 2	Normal range
Blood cell counts			
White blood cells	7.26	8.13	4.19–9.43 10e9/L
Neutrophils	2.2	4.22	1.82–7.47 10e9/L
Lymphocytes	4.09 (H)	2.9	1.16–3.33 10e9/L
Monocytes	0.73 (H)	0.76 (H)	0.19–0.72 10e9/L
Platelets^a^	118 (L)	154	130–400 10e9/L
Mean platelet volume	9.8	10.3	8–12 fL
Hemoglobin	130	149	105–150 g/L
Immunoglobulins			
IgA	0.81	1.35	0.52–1.92 g/L
IgM	0.63	1.07	0.47–3.11 g/L
IgE^b^	423	568	<629 kU/L
IgG^c^	8.92	9.56	7–15.9 g/L
IgG1	5.77	7.54	3.15–8.55 g/L
IgG2	0.30 (L)	0.65	0.64–4.95 g/L
IgG3	0.3	0.29	0.23–1.96 g/L
IgG4	0.158	0.384	0.11–1.57 g/L
Specific antibody titers to diphtheria, tetanus, pneumococcal, rubella, varicella, and measles vaccine antigens	Good, sustained, protective antibody titers to vaccines except borderline measles IgG response	Good, sustained, protective antibody titers to all vaccines	
B cell proliferation (% of CpG-stimulated cells divided)	68.1%	65.0%	63.2–100%^d^
T cell proliferation: mitogen and antigen stimulation of PBMCs by PHA, ConA, PWM, anti-CD3, anti-CD3 + IL-2, IL-2, tetanus toxoid, diphtheria toxoid, and *Candida albicans* antigens	Normal lymphocyte proliferation to all mitogens and antigens tested	Normal lymphocyte proliferation to all mitogens and antigens tested	
NK cell function			
NK cell cytotoxicity (NK cell killing activity)	Normal	Normal	
Degranulation (CD107a^+^)	27%	22%	11–35%
Neutrophils NBT reduction	Normal oxidative burst of 99%	Normal oxidative burst of 100%	
Complement			
CH50 classical	93	70	42–96 U/mL
C3^e^	1.54	1.63	1.1–1.8 g/L
C4^e^	0.26	0.24	0.17–0.39 g/L
MBL	0 (L)	0 (L)	30–200%
Alternate complement	119	107	>40%
Inflammatory markers			
ESR	18 (H)	2	0–9 mm/h
CRP	24.6 (H)	6 (H)	0–5.0 mg/L
Ferritin	400 (H)	2006 (H)	5.5–67 mcg/L
IL-18	> 36,600 (H)	28,803 (H)	<266 pg/mL
SAA	9837	8511	ng/mL, within 30–70% of normal
Auto-antibodies^f^			
ANA	ANA IIF + (1:320, speckled pattern)	All negative	
ENA, anti-TTG-IgA, anti-cardiolipin, anti-B2GP1, LA, ASMA, APCP, anti-LKM, anti-PR3, anti-MPO, anti-CCP, RF, anti-TPO	anti-TPO +		
Lipid profile			
LDL	ND^g^	3.08	Acceptable<2.85 H>3.36 mmol/L
HDL	0.79 (L)	1.2	Acceptable>1.17 mmol/L
TGA	6.1 (H)	2.21 (H)	Acceptable<1.02 H>1.46 mmol/L
Cholesterol	6.86 (H)	5.28 (H)	Acceptable<4.40 H>5 mmol/L

L, low; H, high; PHA, phytohemagglutinin; ConA, concanavalin A; PWM, pokeweed mitogen; NK, natural killer cells; MBL, mannose-binding lectin; ESR, erythrocyte sedimentation rate; CRP, C-reactive protein; SAA, serum amyloid A; pc, percentile; ANA, antinuclear antibodies; ENA, extractable nuclear antigen antibodies; SS-A/B, Sjögren's-syndrome-related antigen A/B autoantibodies; anti-TTG-IgA, anti-tissue transglutaminase IgA antibodies; anti-B2GP1, anti-beta-2-glycoproteins antibodies; ASMA, anti-smooth muscle antibodies; ACPA, anti-citrullinated protein antibodies; anti-LKM, anti-liver-kidney-muscle antibodies; anti-MPO, anti-myeloperoxidase antibodies; anti-CCP, anti-cyclic citrullinated peptide antibodies; RF, rheumatoid factor; anti-TPO, anti-thyroid peroxidase antibodies; LDL, low-density lipoprotein; HDL, high-density lipoprotein; TGA, triglycerides

^a^Thrombocytopenia reported in literature [1]

^b^Increased serum IgE levels reported in literature [3]

^c^Hypergammaglobulinemia reported in literature [2]

^d^Mean ± 2SD, n = 6 healthy donors

^e^Hypocomplementemia (C3 and C4) reported in literature [3]

^f^Positive ANA, anti-dsDNA, anti-ENA (anti-Ro), anti-Sm, and anti-chromatin have been found in individuals with prolidase deficiency even in the absence of clinical findings of SLE [2]

^g^Not determined due to high TGA

**Table 3:** Absolute counts and frequencies of CD4^+^ T cell, CD8^+^ T cell, and CD4^+^ T_reg_ cell subsets in the peripheral blood of healthy controls and PD patients

Cell type	Subset	Patient 1	Patient 2	Normal age-related range (clinical laboratory)
Lymphocytes	Absolute CD3^+^	2394	2830	850–3200 cells/µL
	Absolute CD3^+^CD4^+^	1406	2065	400–2100 cells/µL
	Absolute CD3^+^CD8^+^	1178	766	300–1300 cells/µL
	CD4^+^/CD8^+^ ratio	1.1 (L)	2.5	1.5–2.5
	B cells (CD19^+^)	122	266	120–740 cells/µL
	% class-switched memory B cells	2% (L)	4% (L)	9–26%
	NK cells	1102 (H)	133	7–480 cells/µL
	CD3^+^CD4^–^CD8^–^ alpha/beta	0.6%	0.8%	<1.5%
				Normal range *
CD4^+^ T cells	CD4^+^ memory (CD45RO^+^)	33.1	19.1 (L)	23.4–66.5
	CD4^+^ T_EM_	21.5	11.4	9.1–48.4
	CD4^+^ T_CM_	9.0 (H)	5.3	2.1–7.5
	CD4^+^ T_EMRA_	2.1 (L)	1.8 (L)	3.0–13.6
	CD4^+^ T_eff_	0.3	0.5	0–2.5
	CD4^+^ naïve (CD45RA^+^)	59.1	74.2 (H)	21.2–62.7
CD8^+^ T cells	CD8^+^ memory (CD45RO^+^)	77.3 (H)	39.4	8.2–68.3
	CD8^+^ T_EM_	30.0	14.7	0–30.7
	CD8^+^ T_CM_	2.6 (H)	0.7	0.1–2.5
	CD8^+^ T_EMRA_	43.6 (H)	20.7	8.7–29.0
	CD8^+^ T_eff_	2.4	2.5	0–29.4
	CD8^+^ naïve (CD45RA^+^)	15.2	54.6	8.3–83.9
T follicular cells	CD4^+^ CXCR5^+^	1.8 (L)	2.0 (L)	2.2–9.4
	CD8^+^ CXCR5^+^	0.3	0.2	0–4.4
T_reg_ cells	Total T_reg_ (% of CD4^+^)	3.6	2.8	2.6–6.3
	Naive T_reg_ (CD45RO^–^)	27.1	38.9	15.1–49.4
	Memory T_reg_ (CD45RO^+^)	57.2	41.8	40–82
T cell cytokines	CD4^+^ T_EM_ IFN-γ^+^	37.5	9.3 (L)	12.8–56.4
	CD4^+^ T_EM_ IL-13^+^	3.0	6.8	2.3–7.0
	CD4^+^ T_EM_ IL-17^+^	1.8	2.4	1.0–3.2
	CD8^+^ T_EM_ IFN-γ^+^	55.2	35.5 (L)	42.0–100
	CD8^+^ T_EM_ IL-13^+^	0.7	0.4	0.2–8.1
	CD8^+^ T_EM_ IL-17^+^	1.7 (H)	1.4 (H)	0–0.9
Checkpoint molecules	CD4^+^ T_EM_ CTLA-4^+^	22.4 (L)	21.2 (L)	24.5–43.7**
	CD4^+^ T_EM_ PD-1^+^	58.5	44.9	26.6–70.3
	CD4^+^ T_EM_ TIM-3^+^	3.7 (H)	4.8 (H)	0.4–1.8
Activation marker	CD4^+^ T_EM_ HLA-DR^+^	20.2 (H)	10.2 (H)	1.5–9.4
	CD8^+^ T_EM_ HLA-DR^+^	6.3	7.5	0–22.5

Information on the optimized antibody panels used for flow cytometry can be obtained by contacting the corresponding author. Numbers represent the percentage of cells comprising each subset or percentage of cells expressing the indicated markers.

T_CM_, central memory T cells; T_eff_, effector T cells; T_EM_, effector memory T cells; T_EMRA_, T cell re-expressing CD45RA; H, higher than normal range; L, lower than normal range

*The normal range is represented by mean ± 2 × standard deviation of n = 7 healthy controls, mean age ± SD 34.3 ± 4.9 years and ** mean age ± SD 54.8 ± 10.8 years. T_reg_ cells were defined as CD3^+^CD4^+^CD127^low/–^CD25^high^FoxP3^+^.

Detailed immunophenotyping of T, B, and NK lymphocyte subsets and B cell function were further characterized by flow cytometry (BD LSR Fortessa SORP flow cytometer). Heparinized blood was collected from Pt1, Pt2 at 16 years of age, and healthy, adult volunteers (n = 14, mean age ± SD 45.8 ± 13.2 years, 8 females and 6 males), with no history of infections or autoimmune disease. Peripheral blood mononuclear cells (PBMCs) were isolated using Ficoll-Paque PLUS (GE Healthcare) and cryopreserved in liquid nitrogen. To evaluate B cell function, patient PBMCs were labelled with CellTrace and stimulated with the Toll-like receptor 9 agonist CpG-oligodeoxynucleotide. After 6 days, flow cytometric analysis revealed B cell proliferation in both patients were within the range observed in healthy donors.

NK and T lymphocyte immunophenotyping revealed normal NK and T cell subsets, including a normal T cell CD4^+^/CD8^+^ ratio and no increase in double-negative T cells (CD3^+^CD4^−^CD8^−^TCRαβ^+^). CD4^+^ and CD8^+^ T cell subsets were identified as previously described [24], based on CCR7 and CD45RA expression: naïve, effector (T_eff_), central memory (T_CM_), effector memory (T_EM_), and effector memory re-expressing CD45RA (T_EMRA_). Both patients had lower frequencies of CD4^+^ T_EMRA_ and CXCR5^+^CD4^+^ T follicular helper (T_fh_) cells compared to healthy controls. Pt1 had a higher proportion of CD4^+^ T_CM_ cells among total CD4^+^ T cells and a higher frequency of CD8^+^ memory T cells, T_CM_, and T_EMRA_, among total CD8^+^ T cells (Table 3). To examine the profile of T helper type 1 (Th1), Th2, and Th17 cells, patient PBMCs were stimulated with PMA and ionomycin for 3 h and intracellular cytokine staining was performed for interferon-γ (IFN-γ), interleukin-13 (IL-13), and IL-17, respectively. Both patients had increased proportions of IL-17^+^CD8^+^ T_EM_ cells, compared to healthy controls. Pt2 also had a lower proportion of IFN-γ^+^ cells among both CD4^+^ and CD8^+^ T_EM_ cells. Compared to healthy controls, measuring expression of the checkpoint molecules TIM-3, CTLA-4, and PD-1 revealed higher TIM-3 expression among CD4^+^ T_EM_ cells and lower CTLA-4 expression in CD4^+^ T_EM_ cells in both Pt1 and Pt2. Analysis of the activation marker HLA-DR showed increased expression on CD4^+^ T_EM_ cells in both patients.

The plasma inflammatory cytokine profile and serum amyloid A (SAA) levels were measured using ELISA and a multiplex immunoassay (Human Cytokine 65-Plex Clinical RUO Discover). SAA and proinflammatory cytokines, including IL-1β, IL-6, IL-17A, and tumor necrosis factor (TNF), were within the normal range (15–97.5% distribution) in both patients, except for plasma IL-18 levels, which were increased more than 100-fold compared to healthy controls.

## Discussion

Individuals with PD experience severe morbidity and early death, usually due to infection. To date, 90 patients with PD have been reported in the literature [3], and nine have passed away with infection as a leading cause. PD patients experience bacteremia, skin infections, and cellulitis, with frequent infection by influenza, *Pseudomonas aeruginosa*, and fungi, the latter of which are infections typically observed in combined or innate immune deficiencies [14, 25]. Decreased neutrophil chemotaxis was reported [26], however we did not assess this in this study. Pt1 and Pt2 presented with recurrent respiratory tract infections with MBL complement deficiency being a possible contributing factor. Moreover, prolidase is required for the maturation of type I IFN receptors [27]. PD patients lack PEPD activity, leading to inhibition of type I IFN receptor-dependent immune responses, which are critical for the amplification of innate immunity and the mobilization of adaptive immunity in response to infection.

CXCR5^+^CD4^+^ T_fh_ cells are specialized cells that provide help to B cells especially during affinity maturation which is essential for high-affinity antibody production and the development of memory B cells [28] Laboratory measured immune abnormalities reported in the literature include elevated levels of IgG, IgA, IgM, and IgE, deficiency of the complement component C1q, and low C3 and C4 complement levels [13, 14, 26, 29]. Hypergammaglobulinaemia is likely secondary to recurrent infections or immune dysregulation [26, 29, 30]. The low proportion of T_fh_ cells in both patients may contribute to low class-switched memory B cells. Despite these observations, specific antibody production was intact as well as B cell proliferation to T cell-independent stimulation. It is possible that low circulating T_fh_ cells may not be reflective of lymph node-resident T_fh_ cell populations as the former have been shown to be phenotypically distinct from their counterparts in the tissue [31]. Indeed, mechanisms of immune deficiency in the patients in the present study do not appear to involve germinal center B cell responses or the development of effective humoral immunity.

Reports of immune dysregulation in PD patients have been published, including a patient with very early onset inflammatory bowel disease [32]. Additionally, association between systemic lupus erythematosus (SLE) and PD has been described [8–10, 12, 14, 25, 33] with common symptoms including anemia, thrombocytopenia, hypergammaglobulinemia, hypocomplementemia, and elevated titers of autoantibodies [2]. Pt1 had ANA positivity and anti-TPO autoantibodies. We focused on immune checkpoint molecules and cytokines expressed among effector memory T cells, since naïve T cells express very low levels in both health and disease. We have observed a deficiency of CD4^+^ T_EM_ CTLA-4^+^cells and borderline low T_reg_ cell frequencies. Although this was relatively modest, even partial CTLA-4 deficiency results in severe autoimmune disease such as in CTLA-4 haploinsufficiency with autoimmune infiltration (CHAI) [34]. In contrast, TIM-3 expression in CD4^+^ T_EM_ cells was upregulated, which has been associated with reduced T cell inflammatory responses, and favorable long-term outcomes in multiple autoimmune diseases. T cell cytokine responses in both patients were normal with the exception of elevated proportions of IL-17^+^ CD8^+^ T_EM_ cells [35]. Pt2, who presented with severe atopy, had low proportions of IFN-γ-producing T cells, a relationship which has been previously shown in patients with atopic disease [36]. Immune dysregulation is further evidenced by the chronically elevated CRP in Pt1 and very high IL-18 levels in both patients suggesting an auto-inflammatory process, like in the *NLRC4* inflammasome-related clinical disease spectrum [37]. Prolidase cleaves di- and tripeptides at the carboxyl terminal of proline resulting in a unique ring structure that prevents proteolysis and maintains the protein’s biological activity [38, 39]. Damaged proline in PD, as endogenous danger signal could lead to dysregulated inflammasome signaling and hyperinflammation. Another possible mechanism linking PD with dysregulated immune activity is through the nuclear factor kappa B (NFκB) transcription factor, which represents a critical network for coordinating inflammatory responses. Prolidase activity is inversely associated with NFκB activity. Intriguingly, the complexes which cooperate in the physiological activation or regulation of NFκB activity vary in their roles depending upon the cell type or signaling pathway involved [40]. This may explain the diverse outcomes observed within and between individuals lacking prolidase. In some immune cells or tissue microenvironments, regulation of NFκB-induced inflammation by prolidase is lost while in others it remains intact through a network of tightly regulated compensatory mechanisms. Additional studies are needed to improve our understanding of the biochemistry of prolidase-dependent regulation of transcription factor activity before mechanisms for immune dysregulation in PD become clear.

Both patients had an abnormal lipid profile, which was unexpected as they had a normal BMI, were on a healthy diet, and had no family history of first degree coronary disease. Both had high cholesterol and LDL levels, hepatic steatosis, with hepatomegaly and elevated liver enzymes, which have not been previously observed in PD patients. Prolidase has been linked to lipid metabolism and may explain the hyperlipidemia findings, therefore indicating a need to monitor lipid levels in PD patients [41]. Intellectual disability has been reported in 71% of patients with PD [13]. The IQ scores reported vary from extremely low to low-average, in patients assessed by the WISC-V scale [3]. IQ scores of Pt1 and Pt2 were within the previously reported average for PD patients. The cause of intellectual disability in PD patients is not clear, but a recent study has proposed that PD may affect synaptic neurotransmission [42]. In addition, the basement membrane of the pial meninges (pBM), which is essential for brain cortical maturation, is predominantly composed of type IV collagen which would be negatively affected by PD due to disruptions in collagen metabolism [42].

Pt1 and Pt2 have a classical presentation of PD including dysmorphic facial features, intellectual disability, chronic, severe skin ulcers of the lower extremities, and telangiectasias. Pt1 presented with a more aggressive inflammatory disease with severe skin manifestations and autoimmunity, while Pt2 presented with more atopy. Different PD phenotypes between siblings have been previously reported and varying environmental factors or epigenetic modulators have been proposed as causative [5, 43, 44].

The treatment of PD is symptomatic and there is no recommended or curative regimen. Topical and systemic treatments have been used with only partial improvement reported. Most treatment strategies aim to replace prolidase or treat and stop ulcerative progression. Topical treatments include proline, growth hormone (GH), and tacrolimus [45–48]. Topical proline ointment has been reported to rapidly reduce ulcer size, especially when combined with a 5% glycine ointment, but has not been shown to prevent new ulcers [2, 44, 47]. GH, LMWH, and collagen cofactor Vitamin C (4 g/kg) have been previously shown to accelerate wound healing but has not been shown to prevent new ulcer development [38, 46, 49]. Vitamin C and LMWH have been shown to reduce symptoms where patients had evidence of thrombosis in the cutaneous microcirculation [2, 38, 44, 49–51], Topical tacrolimus, which is immunosuppressive and anti-inflammatory, was found to be very effective in treating ulcers without causing skin atrophy, however, did not prevent new ulcers [48]. Partially successful treatment with systemic immunosuppressive medications or frequent packed red blood cells transfusions have been also reported [13, 51]. In a previous study of a PD patient, hematopoietic stem cell transplantation (HSCT) was performed with signs of successful engraftment, however, the patient developed a severe infection and unfortunately died [52]. Besides monitoring the physical changes to determine clinical response to variable treatments, more objective methods to determine successful treatment could include measuring proline urine metabolism or erythrocyte prolidase levels [13, 51].

The limitations of this study include the use of peripheral blood mononuclear cells without paired tissue biopsies from sites such as the skin or lymph nodes where the balance of inflammatory and immunoregulatory processes may be different. Moreover, immunophenotyping of patients occurred after Pt1 was offered IVIG as disease activity was high preceding the blood sample collection date, which may have influenced the results. Immunophenotyping data were compared to a limited number of healthy controls which were not matched for age or sex, therefore a larger study is required to replicate these findings.

## Conclusion

Prolidase deficiency is a complex disorder with a broad clinical spectrum of presentations and severity. PD, with its immune components, may be classified as a primary immune regulatory disorder (PIRD). Very high IL-18 plasma levels suggest underlying autoinflammatory processes and novel, targeted therapies should be investigated. We suggest monitoring lipid levels in patients with PD to explore a potential pathogenic link. The proposed combined therapy, including IVIG, LMWH, vitamin C, topical proline, and topical tacrolimus is promising, however, it requires long-term follow-up.

## Data Availability

Data are available upon request from the corresponding author.

## Abbreviations

ANA: Antinuclear antibody
anti-TPO: Anti-thyroid peroxidase
CBCd: Complete blood count and differential
CRP: C-reactive protein
ESR: Erythrocyte sedimentation rate
GH: Growth hormone
HSCT: Hematopoietic stem cell transplantation
IVIG: Intravenous immunoglobulin
LMWH: Low molecular weight heparin
MBL: Mannose-binding lectin
NK: Natural killer
pBM: Basement membrane of the pial meninges
PBMC: Peripheral blood mononuclear cell
PD: Prolidase deficiency
Pt1: Patient 1
Pt2: Patient 2
T_CM_: T central memory
T_eff_: T effector
T_EM_: T effector memory
T_EMRA_: T effector memory re-expressing CD45RA
T_fh_: T follicular helper
Treg: T regulatory
SAA: Serum amyloid A
WISC-V: Wechsler Intelligence Scale for Children: 5th Edition
